# Advances in *Rosetta* structure prediction for difficult molecular-replacement problems

**DOI:** 10.1107/S0907444913023305

**Published:** 2013-10-12

**Authors:** Frank DiMaio

**Affiliations:** a Department of Biochemistry, University of Washington, UW Box 357350, Seattle, WA 98195, USA

**Keywords:** structure prediction, molecular replacement, model building

## Abstract

Recent work has shown the effectiveness of structure-prediction methods in solving difficult molecular-replacement problems. The *Rosetta* protein structure modeling suite can aid in the solution of difficult molecular-replacement problems using templates from 15 to 25% sequence identity; *Rosetta* refinement guided by noisy density has consistently led to solved structures where other methods fail. In this paper, an overview of the use of *Rosetta* for these difficult molecular-replacement problems is provided and new modeling developments that further improve model quality are described. Several variations to the method are introduced that significantly reduce the time needed to generate a model and the sampling required to improve the starting template. The improvements are benchmarked on a set of nine difficult cases and it is shown that this improved method obtains consistently better models in less running time. Finally, strategies for best using *Rosetta* to solve difficult molecular-replacement problems are presented and future directions for the role of structure-prediction methods in crystallography are discussed.

## Abstract 2

Modeling advances using *Rosetta* structure prediction to aid in solving difficult molecular-replacement problems are discussed.

## 1. Introduction

*Rosetta* (Rohl *et al.*, 2004 ▶; Das & Baker, 2008 ▶; Leaver-Fay *et al.*, 2011 ▶) has evolved as a comprehensive tool for protein structure modeling, including tools for *ab initio* structure prediction, protein–protein and protein–ligand docking, loop modeling and structure refinement, as well as for designing proteins with new functionalities. While diverse, the components that unite these disparate protocols are (i) the *Rosetta* energy function and (ii) Monte Carlo and gradient-based methods for exploring protein conformational space. *Rosetta*’s energy function (Kuhlman *et al.*, 2003 ▶) evaluates the physical feasibility of a protein’s conformation and consists of both physical and statistical energy terms; sampling methods aim to find the protein conformation that minimizes this energy function.

Although *Rosetta*’s energy function often shows a funnel to the native conformation (Tyka *et al.*, 2011 ▶), for structures larger than 100 residues complete exploration of protein conformational space is intractable (Bradley *et al.*, 2005 ▶). Previous work has shown that comparative modeling and even *ab initio* modeling using *Rosetta* may be used to solve difficult molecular-replacement problems in cases where any available template structure may not. In several cases protein structures modeled by *Rosetta* were more suitable for molecular replace­ment than were the templates, although the results were inconsistent (Qian *et al.*, 2007 ▶; Das & Baker, 2009 ▶).

However, when experimental data, even sparse or noisy data such as cryo-EM density (DiMaio *et al.*, 2009 ▶) or NMR chemical shift data (Raman *et al.*, 2010 ▶), are available they can dramatically limit the size of the conformational space one has to consider, making previously intractable modeling problems feasible. Recent work has shown that combining *Rosetta*’s comparative modeling with density- and energy-guided refinement may also be used to aid in the solution of difficult molecular-replacement problems with much greater consistency than previous work (DiMaio *et al.*, 2011 ▶). This approach, called *MR-Rosetta*, increases the success rate of molecular replacement when starting from 15–30% sequence-identical templates. This manuscript briefly describes the *MR-Rosetta* rebuilding and refinement process and then introduces improvements to the modeling protocol to better handle misalignments and deviations between the template and target structures. We also describe several improvements aimed at faster model generation. Finally, we test these improvements on a benchmark set of nine molecular-replacement cases.

## 2. Methods

**Figure 1:**
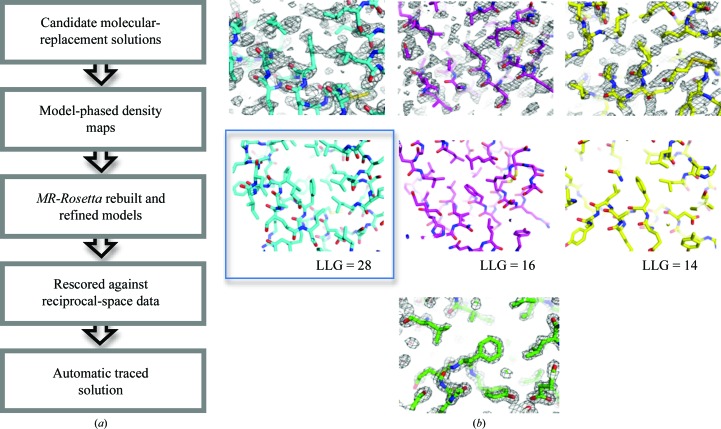
(*a*) An overview of the approach used by *MR-Rosetta* to refine models against noisy density data resulting from difficult molecular-replacement problems. In addition to identifying the correct solution from among a list of candidates, *MR-Rosetta* is often able to improve the model enough so that automatic chain tracing can solve (or very nearly solve) the structure. (*b*) How *Rosetta* combines sequence information to guide backbone sampling with energetics and experimental data during refinement.

An overview of the protocol used by *MR-Rosetta* is shown in Fig. 1 ▶(*a*). The process begins by identifying homologous structures and alignments using *HHsearch* (Söding, 2005 ▶). Threaded models, in which all unaligned residues are removed and non-identically aligned residues are mutated (similar to what is described in Bunkóczi & Read, 2011 ▶), are then generated from an ensemble of homologous structures. Using the molecular-replacement program *Phaser* (McCoy *et al.*, 2007 ▶), we identify a number of potential molecular-replacement solutions; up to five MR solutions from each of up to 20 templates are considered. For each solution, we use the model to phase the data; these maps are used to guide subsequent sampling steps.

**Figure 2:**
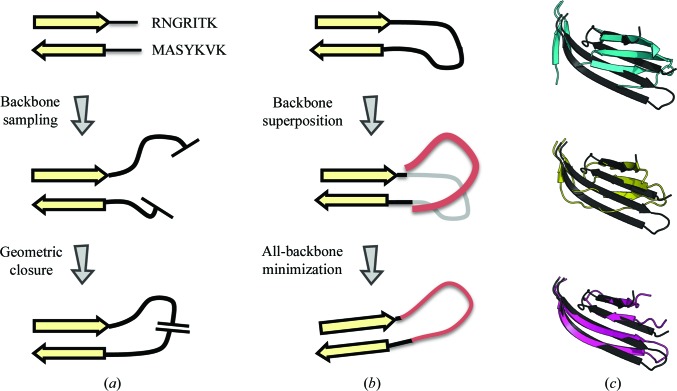
(*a*) An overview of model rebuilding in our previous approach. (*b*) In our new approach, model rebuilding is interspersed with minimization moves, which allow deviations from the template to accommodate the new fragment. (*c*) A brief example of how our improved model building may handle small sequence misalignments. The aligned template (top; cyan) places insertions within a β-strand pairing (native in black). Our previous approach (middle; yellow) breaks the strand pairing. In our new protocol (bottom; magenta), by refining the template backbone during rebuilding the strand pairing is kept intact.

In the next stage, *Rosetta* is used to rebuild gaps in the sequence alignment as well as to refine the entire structure against *Rosetta*’s all-atom energy, guided by the density. The rebuilding step from DiMaio *et al.* (2011 ▶) is illustrated in Fig. 2 ▶(*a*). Rebuilding uses Monte Carlo sampling of backbone ‘fragments’ taken from structures with similar local sequence. Backbone movement propagates to a ‘cutpoint’ and a geometric chain-closure algorithm (Canutescu & Dunbrack, 2003 ▶) was used to create an unbroken chain model. This model is then evaluated against the density and accepted or rejected using the Metropolis criterion; thousands of backbone conformations are sampled this way in a single trajectory. In an *MR-Rosetta* run, all unaligned segments that are eight residues or shorter are sampled in this manner. During this stage, residues aligned to the template (aside from 2–5 residues immediately adjacent to gapped regions) are not allowed to move and each gapped region is sampled independently.

For each candidate MR solution, many (generally tens to hundreds of) conformations are rebuilt and refined. Each solution is then rescored against the unphased crystal data (using *Phaser*’s MR_RNP mode). At this point, if the correct solution is among the initial set it should easily be identifiable by score; also, the model should be sufficiently improved so that interpretation of the model-phased map should be straightforward with automated chain-tracing and refinement programs (see, for example, Terwilliger *et al.*, 2008 ▶; Langer *et al.*, 2008 ▶; Cowtan, 2006 ▶). However, in some cases it still may not be: in these cases iterating reciprocal-space refinement and real-space refinement (in *Rosetta*) may help (as in Valkov *et al.*, 2011 ▶).

Even though the density of the correct solution is often noisy and suffers from model bias, it still contains some information. By refining with *Rosetta*’s physically realistic force field, we are able to improve the fit to the data while maintaining physically favorable structural interactions. While noisy, the density contains sufficient information that it still may be used to restrict conformation space during sampling. The combination of two independent sources of information, the energy function’s measure of physical feasibility combined with the experimental density data, often leads to conformations closer to native than the initial model. This improvement is generally good enough to solve the structure, as was shown in previous work (DiMaio *et al.*, 2011 ▶). The remainder of this section discusses recent improvements to this method.

### 2.1. Improved algorithms for model building

One problem with our original approach is the separation of the backbone-rebuilding and all-atom refinement steps. As illustrated in Fig. 2 ▶(*a*), unaligned and missing backbone segments are first rebuilt using a combination of fragment insertion and geometric loop closure. Only when rebuilding is complete is the backbone in the aligned region allowed to move away from the starting conformation. This approach works well when the template structure is accurate over the aligned regions and the sequence alignment is also accurate. Unfortunately, when either of these does not hold *Rosetta* will inefficiently sample conformational space, heavily biasing the search nearby this incorrect conformation. In these cases, significant sampling is required to the improve models at all; in the worst case, *Rosetta* may be unable to improve the initial model. This is particularly troublesome with errors in sequence alignment: insertions or deletions within secondary-structure elements often led to large modeling errors. Additionally, geometric loop closure frequently led to structures with unreasonable backbone geometry; this too was aggravated by errors in sequence alignment and small errors in the template backbone adjacent to these gapped regions.

We have recently developed an alternate strategy that allows movement in the template structure during modeling. An overview of this strategy is shown in Fig. 2 ▶(*b*). There are two key differences compared with our original approach. Firstly, fragment placement is guided not by setting backbone torsions and propagating movement towards a cutpoint (as in Fig. 2 ▶ *a*), but rather by superimposing the fragment over the current backbone conformation. Secondly, the chain-closure step of Fig. 2 ▶(*a*) is now replaced by Cartesian-space minimization of the *entire structure* against a smooth (differentiable) version of the *Rosetta* low-resolution energy function, together with a scoring term that enforces bond geometry.

This low-resolution energy function primarily favors reasonable backbone geometry (with terms enforcing Ramachandran preferences, backbone hydrogen bonding and van der Waals interactions). Since this energy function only approximates each side chain with a relatively soft interaction center, minimization occurs on a smoother energy landscape, allowing larger backbone movements in aligned regions than does minimization against the all-atom energy function. By letting the template backbone move while gaps are rebuilt, we more naturally handle errors in the template, whether owing to alignment errors or to deviations between the template and the target model. Finally, Fig. 2 ▶(*c*) shows an example where this new approach yields a superior model: by letting the residues adjacent to the gaps move, the approach maintains the strand geometry from the template, even though the sequence alignment was in error.

### 2.2. Faster scoring and minimization into density

The original density scoring in *Rosetta* computes a masked correlation over a neighborhood around each residue; this neighborhood mask is updated as the structure refines. We felt that this formulation was advantageous as the sum of local correlations naturally handled areas of weaker and stronger density that might result from a weak initial molecular-replacement solution. However, this formulation is relatively slow; normalization over this constantly changing mask was expensive computationally. By instead performing an unmasked correlation, we compute the product ρ_calc_·ρ_obs_ for a single atom over the entire map *via* Fourier-space convolution (at dense grid sampling) and use tricubic spline interpolation to very quickly approximate these values and gradients for each atom in the protein during a refinement trajectory (similar to the formulation of Chou *et al.*, 2013 ▶). This offers a significant speedup for full-atom refinement, where thousands of score-function evaluations are required.

### 2.3. Automatic generation of fragments

A second time-consuming step in *MR-Rosetta* is generating backbone fragments, which model the conformational diversity of the backbone given the local sequence. These fragments are used to guide sampling of unaligned regions of the protein (Figs. 2 ▶ *a* and 2 ▶ *b*). *MR-Rosetta* makes use of the same fragment generation as used in *Rosetta* *ab initio* prediction (Gront *et al.*, 2011 ▶), where fragments are chosen using local profile–profile alignments. For many modeling tasks, such as *ab initio* structure prediction, fragment-generation time is inconsequential compared with the time spent sampling, as fragments only need be generated once for each modelled sequence. However, when guided by density, modeling typically converges quickly and models that improve the phasing enough to solve the structure may be generated in as few as 20 Monte Carlo trajectories (Terwilliger, DiMaio *et al.*, 2012 ▶). In these cases, the ∼1 h fragment-generation time may represent a significant fraction of the overall runtime of the modeling pipeline.

To handle this, we have added to *MR-Rosetta* the ability to quickly generate profile-free fragments on the fly. This protocol simply computes a BLOSUM-weighted (Henikoff & Henikoff, 1992 ▶) distance between source and target sequence and selects the best-scoring fragments under this metric. This offers a fairly significant time reduction, taking only 10 s or so per fragment, reducing the time required for fragment generation of an ∼200-residue protein from about an hour to a few minutes. This time reduction comes with some cost: in some cases it may reduce the accuracy of the fragments generated. The average r.m.s. deviation between predicted fragments and the native backbone is higher when fragments are generated from a single sequence than when using profile and predicted secondary-structure information (Gront *et al.*, 2011 ▶).

## 3. Results

A benchmark set consisting of nine of the blind cases from the previous *MR-Rosetta* study was used. These nine were chosen to be of a size that allowed a reasonable testing time and had starting models that made them medium to high difficulty (based on model–map correlation). In these cases, one of the correct initial molecular-replacement hits was chosen and used to phase the data. In each case, a 2*mF*_o_ − *DF*_c_ map was built with *phenix.maps* (Adams *et al.*, 2010 ▶) and models were rebuilt and refined in *Rosetta*. For each of the nine targets, 200 models were generated using the old protocol and 200 with the new protocol. For the 400 models, we computed model density and took the correlation between model density and the density from the final refined structure using *phenix.get_cc_mtz_pdb* using a high-resolution limit of 3 Å (no reciprocal-space refinement was performed prior to computing correlations). We then compared the distribution of correlations from the final models with the starting structure.

**Figure 3:**
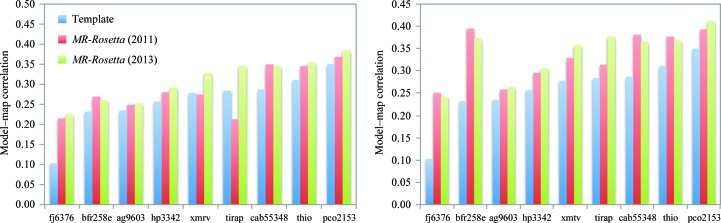
A comparison of the previous and new model-building approaches in *MR-Rosetta*. Plots show the density correlation between models and the 2*mF*_o_ − *DF*_c_ density from the final refined structure, where the models are either the template, the *MR-Rosetta* model using the previous model-building approach or the *MR-Rosetta* model using the new model-building approach. The left plot compares the average model quality, while the right plot shows the selected model quality. Both plots show correlations after one round of model building without reciprocal-space refinement. While most cases show similar performance, there are three cases in which off-template movement allows more accurate model rebuilding.

The results are summarized in Table 1 ▶ and Fig. 3 ▶. Fig. 3 ▶ compares the correlation of the *average* sampled model (left) for the two methods, as well as the *selected* model (right). The selected model reports the top correlation of the best five models according to the *Phaser* LLG (McCoy *et al.*, 2007 ▶). Table 1 ▶ also reports the correlation of the best sampled model of all 200. Our results show a significant improvement (>0.05 increase in average model correlation) in two of these cases, both by average sampled correlation as well as selected model correlation. There is a minor improvement in two other cases (0.01–0.05 increase in average correlation). In all other cases the correlation of the average sampled model is within 0.01 of the previous version. In the two cases where we see significant improvement, there are portions of the template structure that align to the target sequence but move significantly in the final model.

**Figure 4:**
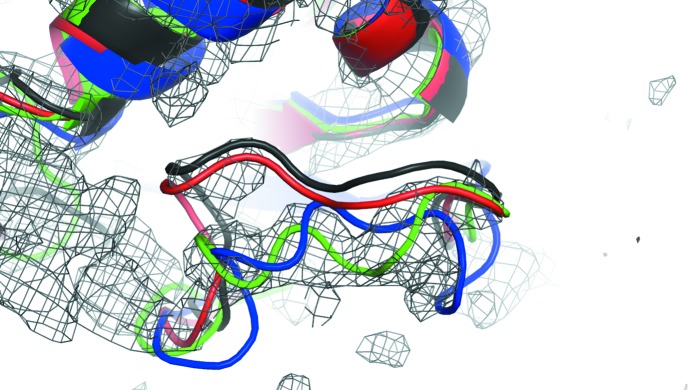
An example illustrating the improvements allowed by the new model-building approach. The template model, indicated in black, has a loop whose conformation is changed in the final model. The previous model-building approach (in red) was unable to move this loop. Our new approach (in green) correctly rebuilds this region, giving better agreement with the final structure (PDB entry 2y92, shown in blue; Valkov *et al.*, 2011 ▶).

Fig. 4 ▶ highlights one of these cases (‘tirap’ in Table 1 ▶ and Fig. 2 ▶). Here, there is a large movement between the template and the final structure in one of the template loops. Our previous protocol, which only allows the backbone to move in regions aligned to the template during all-atom refinement, is unable to correct the model. Our new approach, which lets the template backbone minimize during rebuilding, places the loop in a largely correct conformation. The figure shows only the selected model. However, this movement consistently occurs in independent modeling trajectories (as indicated by the large improvement in the average sampled model correlation).

**Figure 5:**
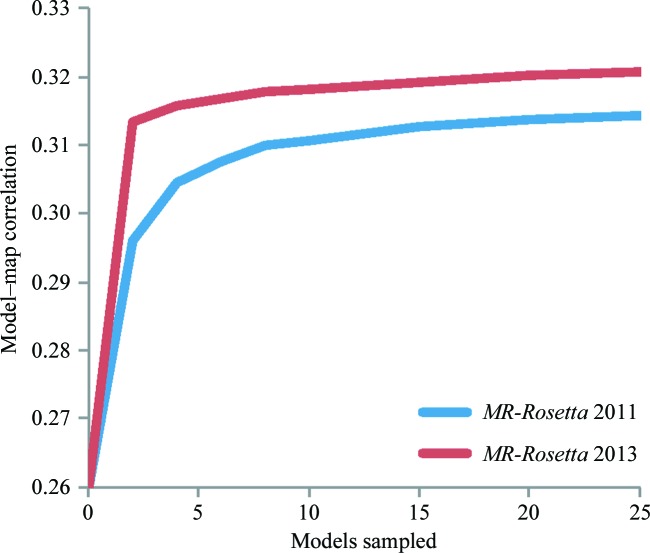
A plot illustrating the role of sampling in improving input models. The correlation of the selected model is plotted as a function of the number of models generated. This plot shows that with the algorithmic improvements 5–10 models are sufficient to see most of the improvement of a large-scale run.

This improved average sampling corresponds to a reduction in the amount of sampling needed in order to obtain a solution of high accuracy. Fig. 5 ▶ computes the correlation of the average selected models as a function of the models generated. The average correlation after *N* samples is provided as *N* ranges from 1 to 40 (above this value, the curve becomes very flat). As the plot shows, our new approach performs significantly better with fewer models generated. On average, using the new protocol, only five models are needed to achieve an average correlation within 0.02 of the correlation achievable with the full 200-sample ensemble.

We also tested our improved sampling algorithms with the two speedups described in the previous section. The results are summarized in Table 1 ▶. As in the previous experiment, 200 models were generated for each of the nine cases. The first of these is faster density scoring, which decreases the average time needed to sample a single model for a 100-residue protein from ∼800 to ∼250 s. As shown in the table, this faster density scoring slightly improves the average sampled correlation over the control run; however, the correlation of the best selected model shows a slight decrease compared with the baseline. However, for large systems, where the sampling time of a single model may be prohibitively expensive, this variant may prove valuable; it is accessed by adding the flag -MR::fast to the standard *MR-Rosetta* command line (see Appendix *A* [App appa] for complete command lines).

The second variant tests our faster fragment-generation method, where sequence alone is used to quickly generate backbone conformational samples rather then the expensive sequence profile-based approach of the previous protocol. All other steps of the algorithm remain unchanged. As Table 1 ▶ illustrates, this new method of fragment generation also shows no loss in final model accuracy. This may not be too surprising as, unlike in *ab initio* prediction, fragments in *MR-Rosetta* are primarily used to sample the conformation of relatively short backbone segments, where the geometry of the adjacent backbone residues makes sampling very constrained. Thus, a small decrease in fragment accuracy may not affect the sampling to an appreciable degree.

## 4. Discussion and conclusions

We have described several modifications to *MR-Rosetta* that allow improved model building and refinement against density data resulting from weak molecular-replacement solutions. The key improvement of our new approach is refinement of the template backbone during the rebuilding stages, which is better suited for handling large movements of the template backbone, as well as smoothly resolving small alignment errors. In cases where the template and sequence alignments are reasonably accurate, we see minimal improvement in the generated models; in other cases we see large improvements. We also introduce two improvements to *MR-Rosetta* that allow much faster model generation without reducing model quality.

Our new rebuilding method offers two key advantages compared with our previous approach. Firstly, by allowing low-resolution minimization during rebuilding, the template may move to better satisfy the density data. The low-resolution energy function in this stage presents fewer energetic barriers as we move the backbone to better satisfy the density. Secondly, minimization allows residues adjacent to those being rebuilt to move slightly, which leads to less strained loop geometry (better Ramachandran probabilities and reduced out-of-plane peptide-bond movement) compared with the geometric closure of our previous approach. Finally, both speedups that we have introduced show no loss (or minimal loss) in model accuracy with significant runtime savings.

A weakness of our approach is that template-backbone movement is still handled largely though minimization, making the method susceptible to becoming stuck in local minima. However, recent work by Terwilliger, Read *et al.* (2012 ▶) escapes local minima in density refinement by explicitly searching local neighborhoods in the density and using these results to perturb the structure. It should be straightforward to make use of this strategy in *MR-Rosetta*: the search directions resulting from this local search can be incorporated as restraints during standard *MR-Rosetta* refinement. This tool should be complementary to *MR-Rosetta* refinement; the *Rosetta* energy function maintains a physically realistic model, while the local density search would pull the model out from local minima.

The improvements in modeling into density resulting from distant molecular-replacement solutions outlined in this paper should serve to further increase the radius of convergence of molecular replacement. By better handling small errors in sequence alignment, the new approach should improve the modeling of molecular-replacement solutions with very low sequence identity (15% or lower). These advancements, along with those proposed, should allow even greater application of these methods in solving crystallographic data sets.

## Figures and Tables

**Table 1:** A summary of the results for all data sets in the benchmark For each variation of the approach, we report (out of 200 generated structures) the correlation of (i) the average model (Avg. CC); (ii) the best sampled model (Best CC); and (iii) the best selected model (Sel. CC), where five models are selected using the *Phaser* LLG. Bold values indicate the highest correlation for each target. While fast density scoring gives the best models by average and the best correlation, it generally has correlation with the selected model.

	Input	*MR-Rosetta* 2011			*MR-Rosetta* 2013			*MR-Rosetta* 2013 with faster density scoring			*MR-Rosetta* 2013 with faster fragment generation		
	CC	Avg. CC	Best CC	Sel. CC	Avg. CC	Best CC	Sel. CC	Avg. CC	Best CC	Sel. CC	Avg. CC	Best CC	Sel. CC
fj6376	0.103	0.215	0.257	0.251	0.225	0.259	0.242	**0.230**	**0.263**	0.249	0.223	0.256	**0.256**
bfr258e	0.232	**0.269**	**0.395**	**0.395**	0.261	0.373	0.373	0.265	0.365	0.306	0.265	0.382	0.375
ag9603	0.235	0.249	0.268	0.258	**0.252**	**0.279**	**0.264**	0.250	0.276	0.248	**0.252**	**0.279**	**0.264**
hp3342	0.257	0.281	0.317	0.296	0.292	0.312	0.306	**0.301**	**0.320**	**0.314**	0.289	0.311	0.300
xmrv	0.278	0.275	0.330	0.329	0.328	0.364	0.358	**0.335**	**0.380**	0.363	0.334	0.379	**0.373**
tirap	0.284	0.213	0.331	0.314	0.345	0.385	0.377	**0.349**	0.384	0.374	0.343	**0.403**	**0.403**
cab55348	0.287	0.350	0.384	0.381	0.346	0.371	0.366	**0.357**	**0.384**	0.371	0.344	0.373	**0.373**
thio	0.311	0.345	0.382	0.377	0.355	0.373	0.369	**0.359**	**0.383**	**0.383**	0.351	0.376	0.362
pc02153	0.350	0.368	0.397	0.393	0.385	0.412	0.412	**0.394**	**0.417**	**0.416**	0.363	0.403	0.392
Mean	0.260	0.285	0.340	0.333	0.310	0.348	0.341	0.316	0.352	0.336	0.307	0.351	0.344

## Appendix A *MR-Rosetta* command lines

*MR-Rosetta* is available from http://www.rosettacommons.org and is free to academic users. The methods described in this paper are available beginning with version 3.6 or weekly releases after 1 September 2013.

The command line used to run default *MR-Rosetta* (2013) is

To enable faster evaluation of density agreement, the flag -­MR::fast is added to the command line and -edensity:grid_spacing 1.5 should be changed to -edensity:grid_spacing 1.0.

Finally, to enable automatic fragment generation, simply omit the line -loops::frag_files. These two options may be used together.
